# Inequalities in effective coverage measures: are we asking too much of the data?

**DOI:** 10.1136/bmjgh-2022-009200

**Published:** 2022-05-24

**Authors:** Josephine Exley, Tanya Marchant

**Affiliations:** Department of Disease Control, London School of Hygiene & Tropical Medicine, London, UK

**Keywords:** Health services research, Health systems evaluation, Maternal health, Child health, Community-based survey

**Summary box**

- The need to shift to effective coverage measures has gained widespread acknowledgement. Effective coverage combines need, use and quality of care into a single metric to estimate the proportion of a population in need of a service that resulted in a positive health outcome from that service.
- To support efforts towards universal health coverage, effective coverage measures should assess inequalities. At present, direct measures of equity, such as wealth, age, ethnicity, gender, education, place of residence, are only available in household data. However, population-level data alone do not provide information on all components of quality of care and may have poor validity.
- Many measure of effective coverage require linking household data with information from health facilities on the quality of care provided. Health facility data provide a summary of quality of care at the facility-level and consequently ignores variation that may exist between service users with different characteristics.
- Inequalities in effective coverage may be larger than we are able to demonstrate using existing data sources most commonly used to construct effective coverage measures.

Effective coverage measures combine need, use and quality of care into a single metric to estimate the benefit of a service or intervention. Effective coverage is defined as the proportion of the population in need of a service that resulted in a positive health outcome from that service.^1^ For reproductive, maternal, newborn, child health and nutrition (RMNCH+N) services and interventions, effective coverage can be defined using a cascade (see figure 1). Effective coverage is represented by the final step of the cascade, while the full cascade can be used to identify bottlenecks in implementation.

**Figure 1:**
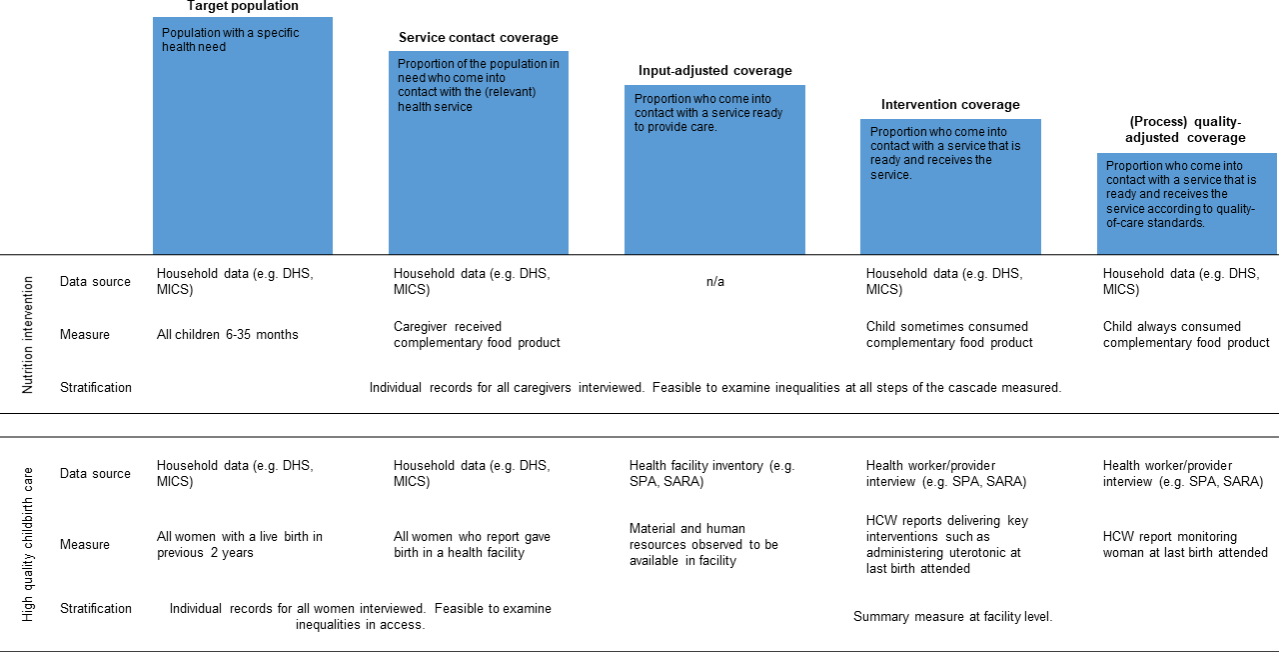
Overview of the data used to measure each step of the coverage cascade* and the stratification possible for a complementary feeding intervention and childbirth care. *Adapted from Marsh *et al*.^1^ DHS, Demographic and Health Surveys; MICS, Multiple Indicator Cluster Surveys; SARA, Service Availability and Readiness Assessments; SPA, Service Provision Assessments.

Universal health coverage means that high-quality interventions and services are available to all.^2–4^ Inequalities in the availability and quality of health services exist at all levels: between geographic regions, within geographic regions, and even within individual health facilities and families.^5^ To address inequalities effective coverage measures should be disaggregated by key sociodemographic and economic variables^1^—such as wealth, age, ethnicity, gender, education, place of residence.^6^

The potential to investigate inequalities in effective coverage is dependent on the data used to construct each step in the cascade. Here, we illustrate two methodological constraints that limit measuring inequalities in effective coverage when using: (1) only population-based data such as Demographic and Health Surveys (DHS) or Multiple Indicator Cluster Surveys (MICS) (eg, complementary feeding interventions) and (2) linked population and health facility data such as Service Provision Assessments (SPA) or Service Availability and Readiness Assessments (SARA) (eg, high-quality childbirth care), summarised in figure 1.

## 1. Population-level data alone do not provide information on all quality of care steps of the cascade and may have low validity.

A literature review of effective coverage measures revealed 14 studies that used only population-level data.^7^ A common example was treatment for malnutrition that typically reflected caregiver reports of whether nutritional interventions were received, whether children were ever given nutritional interventions, and whether the interventions were used appropriately in the household (see figure 1). Quality dimensions of health provider practise were not incorporated.

Since information on sociodemographic and economic variables is typically captured in household surveys, it is possible to stratify each of the relevant steps of the cascade by the desired measure of equity. However, household data provide no information on inputs and evidence on the validity of coverage data collected through household surveys suggest that while it can provide accurate coverage measures for some interventions, for many interventions household respondents cannot accurately report on quality of care dimensions.^8–10^ For the latter, alternative measurement approaches that link multiple data sources have been recommended.

## 2. Facility-level data do not include the individual data needed to track inequalities.

For many services (such as childbirth care), effective coverage measurement relies on linking data on access to care, derived from household surveys, where measures of inequality are incorporated, with information on the quality of care (inputs, interventions, process and experience of care) from health facility datasets.^11–13^ Health facility data, for example, nationally representative surveys such as SPA or SARA or indeed routine data sources such as District Health Information Software 2 (DHIS-2, do not report individual-level data but instead provide a summary of a facility’s capacity to provide high-quality care. Applying a facility-level score to each step of the cascade derived from health data (see figure 1), assumes that there are no systematic differences in the quality of care between individuals attending the same facility. However, evidence demonstrates that this is not the case; individuals with different characteristics receive different quality of care.^5 14^ Estimates of inequalities in effective coverage measures that are derived from linked household and facility data are driven only by the access to care measure.

There are further implications depending on the method applied for linking household and health facility data, whether: (1) individual or exact-match linking or (2) ecological linking.^11–13^

Exact-match linking of individuals in population data to the exact health facility they attended will capture systematic differences in care-seeking behaviour between individuals with different characteristics—for example, that wealthier individuals are more likely to bypass their nearest sources of care to seek higher quality care—either outside of their catchment area or at a higher level facility.^5 15^ Ecological linking—in which individuals from population data are linked to an average quality score across multiple health facilities—takes us a step further away, since it assumes there are no systematic differences in care-seeking behaviour between individuals with different characteristics. Adjusting for the type of facility that people report receiving care from has been demonstrated to generate valid measures of effective coverage, as likely accounts for some difference in care-seeking behaviour.^12 13^ Even so the approach ignores intersectionality and assumes that the quality and experience of care is homogeneous across facilities included in the average score, that is, that the average quality of primary healthcare facilities accessed by the wealthiest people is the same as the average quality of primary healthcare facilities accessed by the poorest people.

Herein lies the measurement dilemma. Relying on summary facility measures for linked effective coverage ignores variation in quality of care both within and (where using ecological linking approaches) between facilities. While generating effective coverage measures using only household data limits the adjustment made for quality and introduces issues with the reliability and validity of measures. In both scenarios, inequalities in effective coverage are driven only by the steps that use population data and are likely to be underestimated as a result. It is important to be mindful of which stratified analyses are feasible and what they are able to tell us about inequalities in effective coverage and refrain from asking too much of the data.

Effective coverage measures remain a crucial tool as we move towards universal access to high-quality care; we need to adjust coverage measures for the process and experience of care for individuals. Alongside continuing to promote effective coverage, we need to support the adoption of unique health identifiers that would allow us to link information on individuals’ care-seeking with information on the quality of care received. In the meantime, greater advocacy and investment in health information systems is needed to shift from reporting aggregated-level to individual-level data and to capture information on individual patients that would enable examination of inequalities within facilities.

## Data Availability

There are no data in this work.
